# Complications after surgical treatment of pelvic fractures: a five-year follow-up of 194 patients

**DOI:** 10.1007/s00590-022-03215-0

**Published:** 2022-02-10

**Authors:** Natalie Lundin, Anders Enocson

**Affiliations:** 1 Department of Molecular Medicine and Surgery, Karolinska Institute, Stockholm, Sweden; 2 Department of Trauma, Acute Surgery and Orthopaedics, Karolinska University Hospital, Stockholm, Sweden

**Keywords:** Pelvic fracture, Trauma, Epidemiology, Surgical treatment

## Abstract

**Purpose:**

Surgical treatment of pelvic fractures is an advanced intervention associated with multiple complications. The primary aim of this study was to investigate the rate of unplanned reoperations after pelvic fracture surgery. Secondary aims included occurrence of other adverse events and mortality.

**Methods:**

All adult patients ≥ 18 years with surgically treated pelvic fracture operated at the Karolinska University Hospital in Sweden between 2010 and 2019 were identified and retrospectively included. Data were collected through review of medical records and radiographs. Logistic regression analysis was performed to evaluate factors associated with unplanned reoperations and other adverse events.

**Results:**

A total of 194 patients were included with mean age (± SD, range) 45.4 (16, 18–83) years. 62% were males (*n* = 121) and the median (IQR) follow-up time was 1890 (1791) days (4.9 years). Forty-eight patients (25%) had an unplanned reoperation, with infection being the most common cause of reoperation (*n* = 18, 9.3%). Seventy-eight (40%) patients had an adverse event not requiring reoperation and the most common event was nerve injury (*n* = 34, 18%). Concomitant abdominal injury was identified as a risk factor for an adverse event (OR 2.5, 95% CI 1.3–4.9, *p* < 0.01). 30-day mortality was 1.5% and 1-year mortality 6.2%.

**Conclusion:**

The rate of unplanned reoperation after pelvic fracture surgery was high, as was the rate of other adverse events not requiring surgery. No identified risk factor was found to predict further surgery, but concomitant abdominal injury was a risk factor for other adverse events. Mortality was low at both 30 days and 1 year.

## Introduction

High-energy pelvic fractures are rare orthopedic injuries often encountered among multi-traumatized patients [1]. Although not an everyday fracture, these patients demand prompt and accurate care starting in the emergency room with sometimes lifesaving primary measures until the definitive treatment of the fracture, which often includes advanced surgery [2]. Complications after pelvic surgery are commonly seen and ranges from infections, thromboembolic events to unplanned reoperations and death [3–5].

Both the incidence of pelvic fractures and the rate of surgical treatment has recently been shown to increase [6, 7]. There is a wide range of surgical methods to restore the anatomy and stability of the pelvic ring, with the prevailing method of today being internal osteosynthesis rather than the use of external fixators [8, 9]. Hardware related complications are previously described and certain surgical elements, like percutaneous sacroiliac (SI)-screw placement are considered particularly challenging [10]. Earlier studies on reoperation rates after pelvic fracture surgery are scarce, but existing data report unplanned reoperations in 15–22% of treated patients, with even higher rates for subgroups of patients with high-grade instability fractures [3–5, 11, 12]. Factors associated with increased risk for reoperation are not properly explored previously but increasing fracture complexity and associated visceral injuries have been proposed [11]. The total complication rate including all adverse events is even less well described in unselected materials of patients with pelvic fracture, but rates as high as 25–30% have been reported [4, 10].

Most existing studies on complications after pelvic fracture surgery investigate isolated subgroups of patients after certain surgical treatments and certain selected complications [10–12]. There is a shortage of long-term follow-up of larger cohorts of patients examining their risk for complication after surgical treatment.

Our primary aim was to investigate the rate of unplanned reoperations after pelvic fracture surgery, and secondary aims were occurrence of other adverse events and mortality.

## Patients and methods

We included all adult patients ≥ 18 years with a pelvic fracture that were surgically treated at the Karolinska University Hospital in Stockholm, Sweden, during a 10-year period (2010–2019). Patients with combined pelvic and acetabular fracture were also included in the study. Non-Swedish residents were excluded due to uncertain follow-up. Patients were selected through the local surgical database and all medical records were manually reviewed.

Collected demographic variables included patient age, gender, and ASA-classification. Injury variables collected were date of injury, injury mechanism, concomitant injuries, vital parameters upon arrival (systolic blood pressure, pulse rate, Glasgow Coma Scale, hemoglobin level), acute pelvic packing, and/or angiography. Fractures were classified according to Young–Burgess [anteroposterior compression (APC), lateral compression (LC), vertical shear (VS)] [13], or as combined (pelvic and acetabular) or as isolated sacral. Fractures were classified preoperatively by the pelvic surgeon performing the operation and confirmed by both authors reviewing the preoperative computer tomography (CT) scans. Treatment variables were date of primary surgery, type of surgical treatment including incision and type of osteosynthesis and/or external fixation. Follow-op variables included any adverse event not requiring surgical treatment (nerve injury, pneumonia, pulmonary embolism, deep venous thrombosis, urinary tract infection, sepsis, kidney failure, superficial wound infection), any unplanned reoperation including causes and types of reoperations and mortality. In addition, length of stay at hospital and intensive care unit (ICU) were recorded.

All patients underwent surgical treatment within 1-month (31) days after injury. Follow-up time was from injury date until 31/12 2020 or death.

### Statistical methods

Numerical data are presented as mean (± SD, range) or median (IQR, range). Categorical data are presented as frequency and percent distribution. Logistic regression analysis was performed to evaluate factors associated with unplanned reoperations and other adverse events. Age, gender, fracture type, and concomitant abdominal injury were tested. First, crude association for each variable was tested in univariable models. Second, a multivariable model was used to study the adjusted associations. The associations are presented as odds ratios (ORs) with 95% confidence intervals (CIs). The results were considered significant at *p* < 0.05. The statistical software used was IBM SPSS Statistics, Version 25 for Windows (SPSS Inc., Chicago, Illinois).

## Results

### Epidemiology

A total of 194 patients with surgically treated pelvic fracture were included. The majority of the patients were male (*n* = 121, 62%), and the mean age (± SD, range) was 45.4 (16, 18–83) years. The median (IQR) follow-up time was 1890 (1791) days (4.9 years). High fall (> 2 m) was the most common (*n* = 70, 36%) mechanism of injury, followed by car accident (*n* = 28, 14%) and motorcycle accident (*n* = 26, 13%). Other vehicle related mechanisms (pedestrian hit by car, cyclist hit by car or snowmobile accident) accounted for 11% (*n* = 21) of the injuries and horse accidents accounted for 10% (*n* = 19), (Table 1). A total of 44 patients (23%) were reported to have an intentional injury mechanism (suicide attempt), and among those a high fall was the dominating mechanism of injury (*n* = 39/44, 89%). Most patients (*n* = 188, 97%) had a high-energy trauma mechanism.

**Table 1:** Epidemiology, vital parameters on arrival, time to surgery, and length of stay

Variable	All patients *n* = 194
Age; mean (± SD, range)	45.4 (16, 18–83)
Age ≥ 60; *n* = (%)	48 (25)
Gender female; *n* = (%)	73 (38)
ASA-class 3, 4; *n* (%)	61 (31)
Injury mechanism; *n* = (%)	
Simple fall	2 (1.0)
High fall	70 (36)
Car related	28 (14)
Motorcycle related	26 (13)
Other vehicle related	21 (11)
Horse related	19 (9.8)
Other	28 (14)
High-energy trauma mechanism; *n* = (%)	188 (97)
Intentional cause of injury; *n* = (%)	44 (23)
GCS; median (IQR)	15 (3)
GCS < 9; *n* = (%)	42 (22)
SBP (mmHg); median (IQR)	120 (31)
Shock; *n* = (%)	29 (15)
Pulse rate; median (IQR)	90 (28)
Hb (g/L); median (IQR)	121 (31)
Head or neck injury; *n* = (%)	63 (33)
Chest injury; *n* = (%)	98 (51)
Abdominal injury; *n* = (%)	50 (26)
Major spine injury; *n* = (%)	43 (22)
Major upper limb injury; *n* = (%)	41 (21)
Major lower limb injury; *n* = (%)	48 (25)
Bladder injury; *n* = (%)	18 (9.3)
Dislocated hip; *n* = (%)	10 (5.2)
Time to surgery (days); median (IQR)	3.0 (3)
Hospital length of stay (days); median (IQR)	15 (23)
ICU care; *n* = (%)	118 (61)
ICU care length of stay: median (IQR)	7.0 (16)

*IQR* interquartile range, *ASA* American Society of Anesthesiologists, *GCS* Glasgow Coma Scale, *SBP* systolic blood pressure, *Hb* hemoglobin, *ICU* intensive care unit

15% of the patients (*n* = 29) exhibited signs of circulatory shock upon arrival with systolic blood pressure < 90 mmHg. 16% (*n* = 32) of the patients underwent acute pelvic packing and 13% (*n* = 26) angiography with or without embolization. 6% (*n* = 12) of the patients were treated with both pelvic packing and angiography with or without embolization.

Associated injuries were common; 51% (*n* = 98) had a concomitant chest injury, 33% (*n* = 63) a head or neck injury and 26% (*n* = 50) an abdominal injury (Table 1).

### Treatment times

The median (IQR) time from injury to surgery was 3 (3) days. 61% (*n* = 118) of the patients needed intensive care and the median (IQR) total hospital length of stay was 15 (23) days (Table 1).

### Fracture classification and surgical procedures

The most common type of pelvic fracture was vertical shear (*n* = 45, 23%), followed by combined (both acetabular and pelvic) (*n* = 37, 19%) and APC type 2 (*n* = 31, 16%). Eight patients had sustained an open fracture. The most common type of osteosynthesis was plate fixation (*n* = 144, 74%) and/or SI-screw (*n* = 100, 52%). Detailed data on fracture types in relation to treatment are presented in Table 2. Four patients were treated with a temporary external fixation, and 22 patients (11%) were treated with final external fixation alone or together with internal osteosynthesis.

**Table 2:** Type of pelvic fracture in relation to treatment

Fracture type; *n* = (%)	Type of treatment; *n* =			
	Plating	SI-screw	Separate screw	Spinopelvic
All; 194 (100)	144	100	38	20
APC1; 2 (1.0)	1	1	0	0
APC2; 31 (16)	24	16	3	0
APC3; 16 (8.2)	12	9	1	1
LC1; 3 (1.5)	3	3	0	0
LC2; 24 (12)	18	11	5	0
LC3; 15 (7.7)	11	13	3	0
Vertical shear; 45 (23)	32	30	5	9
Combined; 37 (19)	34	15	11	3
Isolated sacral; 7 (3.6)	0	2	0	5
Unable to classify; 14 (7.2)	9	0	10	2

*APC* anteroposterior compression, *LC* lateral compression

### Surgical approaches

Surgical approaches used for the pelvic fractures were Stoppa with single (medial) window (*n* = 68), Stoppa with two windows (*n* = 27), Kocher–Langenbeck (*n* = 13), Ilioinguinal (*n* = 12), and/or other incisions (*n* = 92). Other incisions included posterior sacral incision for spinopelvic stabilization, incisions for insertion of external fixator, percutaneous screw placement (not SI-screw), or other type of incision not definable.

### Reoperations

**Fig. 1:**
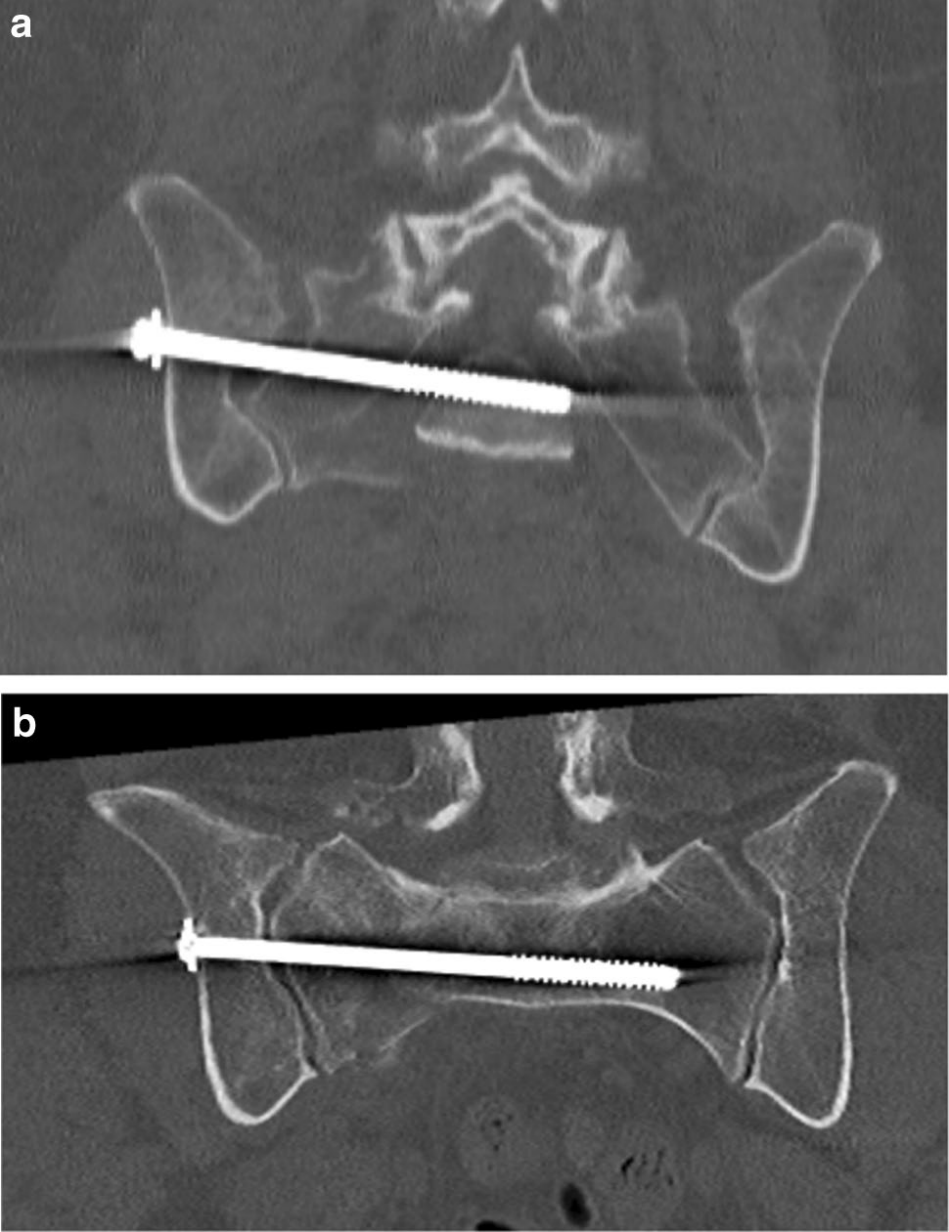
**a** Postoperative CT-scan in a 60-year female with a vertical sheer type fracture displaying an SI-screw penetrating the right and touching the left S1 foramina. **b** Postoperative CT-scan in the same patient after reoperation with exchange of the SI-screw

**Fig. 2:**
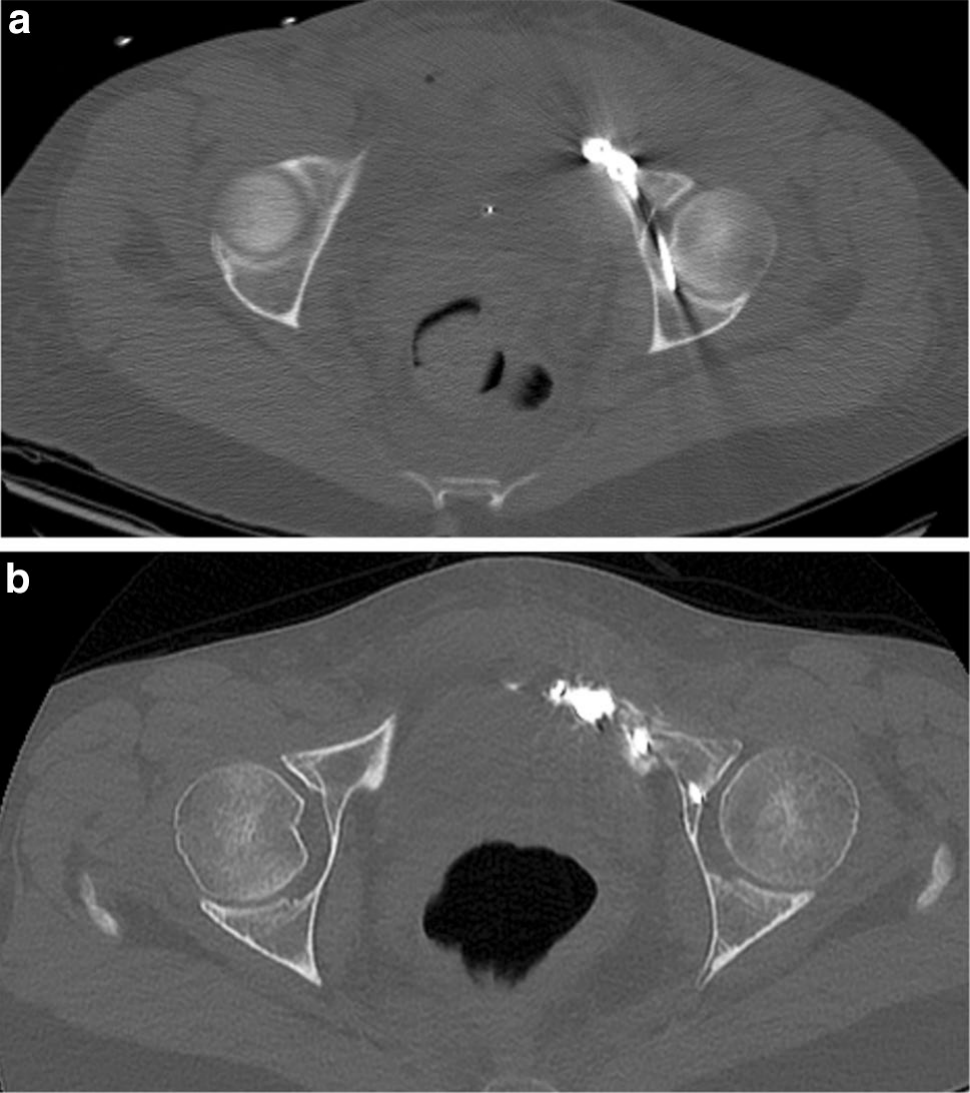
**a** Postoperative CT-scan in a 48-year female with a lateral compression type fracture displaying a screw penetrating the left acetabulum. **b** Postoperative CT-scan in the same patient after reoperation with exchange of the screw

A total of 48 patients (25%) had an unplanned reoperation. The median (IQR, range) time to the first reoperation was 19 (280, 2–1675) days. Infection was the most common cause of reoperation (*n* = 18, 9.3%) followed by malplaced implant and mechanical irritation affecting 11 patients (5.7%), respectively (Table 3). Of the 11 patients with malplaced implant, seven were misplaced SI-screws, two were screws placed intraarticularly, one was an incorrectly placed plate and one incorrectly placed external fixator. CT scans from two of the patients requiring reoperation due to malplaced implants are displayed in Figs. 1a, b and 2a, b. Twenty-four patients (13%) had multiple reoperations, ranging from 2 to 10 additional surgeries. The main reason for these multiple reoperations was infection that required repeated debridement (*n* = 20/24 patients). Four of the 24 patients that underwent > 1 reoperation was deceased within the first year after the initial surgery. In order to evaluate factors contributing with an increased risk for reoperation logistic regression analysis was performed. Age [≤ 45 or > 45 years (median age)], gender (male or female), fracture type (APC, LC, VS, combined isolated sacral, or unable to classify), and abdominal injury (yes or no) were tested. None of the tested variables were associated with an increased risk for reoperation in uni or multivariable analyses.

**Table 3:** Indications for unplanned reoperations

Indication; *n* = (%)	All patients *n* = 194
Infection	18 (9.3)
Malplaced implant	11 (5.7)
Mechanical irritation	11 (5.7)
Failure of osteosynthesis	4 (2.1)
Heterotopic ossification	3 (1.5)
Other	1 (0.5)
All	48 (25)

### Adverse events and mortality

A total of 78 patients (40%) had any kind of adverse event not requiring reoperation. The most common adverse event was nerve injury (*n* = 34, 18%), followed by pneumonia (*n* = 26, 13%) and pulmonary embolism (*n* = 17, 8.8%) (Table 4). In order to evaluate factors contributing to adverse events logistic regression analysis was performed. Age [≤ 45 or > 45 years (median age)], gender (male or female), fracture type (APC, LC, VS, combined, isolated sacral, or unable to classify), and abdominal injury (yes or no) were tested. The presence of a concomitant abdominal injury was associated with an increased risk for an adverse event in both the univariable (OR 2.4, 95% CI 1.2–4.6, *p* < 0.01) and the multivariable (OR 2.5, 95% CI 1.3–4.9, *p* < 0.01) analyses. None of the other variables were associated with an increased risk for an adverse event. The 30-day mortality was 1.5% (*n* = 3) and the 1-year mortality 6.2% (*n* = 12), for all patients.

**Table 4:** Adverse events not requiring reoperation

Adverse event; *n* (%)	All patients *n* = 194
Nerve injury	34 (18)
Pneumonia	26 (13)
PE	17 (8.8)
DVT	11 (5.7)
UTI	10 (5.2)
Sepsis	9 (4.6)
Kidney failure	5 (2.6)
Superficial wound infection	3 (1.5)
All	78 (40)

*PE* pulmonary embolism, *DVT* deep venous thrombosis, *UTI* urinary tract infection

## Discussion

Our main finding was a high rate (25%) of unplanned reoperations after pelvic fracture surgery. No identified predictor for reoperation could be found. The rate of adverse events not requiring reoperation was 40% and concomitant abdominal injury was identified as a risk factor. Mortality among patients with surgically treated pelvic fractures was low.

Other comparable studies on unplanned reoperations with proper follow-up time are mainly lacking but a few studies exist. An American study by Ochenjele et al. [11] investigated a large cohort (913 patients) after surgical treatment of pelvic fracture. They found a much lower reoperation rate (15%) compared to our study but had a considerably shorter follow-up time where only approximately half of their patients were followed > 6 months. Also, they did not include reoperations due to “mechanical irritation of device” as did this study, which could explain their lower rate. Their most common cause for reoperation was infection, as in our study, and with a similar rate of 8% compared to 9.3% in our study. Another American study by Sems et al. [4] examining 182 patients after pelvic fracture surgery, with a minimum follow-up of only 3 months, found a reoperation rate of 16%. They did not however include later reoperations for removal of hardware or heterotopic ossification as we did in our study. The studies by Ochenjele et al. and Sems et al. found reoperation rates due to fixation failure at 6.0 and 9.3%, respectively, comparable to our rate of 7.7%.

Numbers on adverse events not requiring reoperations are slightly harder to interpret and compare. Patients with pelvic fracture, even the ones not treated surgically, have a high rate of sustaining adverse events like thromboembolism and infections [10]. We found a total of 40% of the patients sustaining an adverse event, with the most common being nerve injury (18%). In this broad category all reported postoperative nerve injuries were accounted for, both transient and long-term remaining. Injury to the lateral femoral cutaneous nerve was included, a common negative side effect after pelvic surgery often associated with the former more commonly used ilioinguinal incision. Also, some of the patient’s preoperative nerve function was not documented, and so some nerve injuries may not have been iatrogenic but associated with the initial trauma. Occasional earlier reports on nerve injury report overall lower rates, < 5% [4, 14], but without completely comparable cohorts to our study.

The second most common adverse event not requiring reoperation was pneumonia (13%) and thirdly pulmonary embolism (9%). The previous literature is lacking to compare these numbers adequately, but the results highlight the importance of appropriate comprehensive medical care to prevent and detect these complications early, and late. We could only identify one factor associated with increased risk for an adverse event, and this was concomitant abdominal injury. An earlier study found a correlation between abdominal injury and unplanned reoperation [11], which was not found in this material.

The proportion of patients with combined fracture was somewhat high (19%) but comparable to what Ochenjele et al. found (18%). In opposite to their results, we could not identify this potentially more complex fracture type as a risk factor for unplanned reoperation.

Mortality at 30 days and 1 year was low at 1.5 and 6%, respectively. In-hospital mortality among patients with high-energy pelvic fracture is previously described at rates between 6 and 31% but few studies report on mortality among patients exclusively surgically treated [15–18]. One Canadian study reported 30-day and 1-year mortality rates of 3.0 and 3.8%, respectively, among patients treated with ORIF [19]. Existing studies on Swedish high-energy pelvic fracture patients report mortality rates at 8–9% at 30 days and 9–10% at 1 year [5, 20]. It is of course explainable that severely traumatized patients might die before being candidates to surgery for their pelvic fracture, still the low mortality rates found in our study reflects an overall good treatment and outcome for the surgically treated patients.

### Strengths and limitations

This study investigated a relatively large number of patients treated at a major trauma center in Sweden. A major strength was the long follow-up time, allowing for the capture of late as well as early complications. All reviewing of medical charts and fracture classification was performed by the two authors, assuring consistency in collecting the data. The main limitation of this study was the retrospective design, and we cannot completely guarantee that some patients might have sustained a reoperation or adverse event at another hospital, although care was taken to include these when information was present. Also, the single-center design of the study might limit the generalizability of the results.
